# Human CD8^+^HLA-DR^+^ Regulatory T Cells, Similarly to Classical CD4^+^Foxp3^+^ Cells, Suppress Immune Responses via PD-1/PD-L1 Axis

**DOI:** 10.3389/fimmu.2018.02788

**Published:** 2018-11-29

**Authors:** Andres Machicote, Santiago Belén, Placida Baz, Luis A. Billordo, Leonardo Fainboim

**Affiliations:** ^1^Laboratorio de Inmunogenética, Facultad de Farmacia y Bioquímica, Instituto de Inmunología, Genética y Metabolismo, Universidad de Buenos Aires-Consejo Nacional de Investigaciones Científicas y Técnicas, Buenos Aires, Argentina; ^2^Departamento de Microbiología, Parasitología e Inmunología, Facultad de Medicina, Universidad de Buenos Aires, Buenos Aires, Argentina

**Keywords:** CD8^+^HLA-DR^+^, PD-1/PD-L1, TIGIT, CCR4, CD127, regulatory T cells, anti-PD-1

## Abstract

We have previously identified a human CD8^+^HLA-DR^+^ regulatory T cell subset with the ability to suppress proliferation of autologous PBMCs responder cells through cell contact and CTLA-4 co-inhibitory molecule. The present study characterizes the complete phenotype of CD8^+^HLA-DR^+^ Treg cells which showed great similarities with classical CD4^+^ cells expressing forkhead box P3 (FOXP3). The shared features included the expression of programmed cell death protein 1 (PD-1), T-cell immunoreceptor with Ig and ITIM domains (TIGIT), C-C chemokine receptor type 4 and 5 (CCR4 and CCR5), low expression of CD127, and a memory and effector-like phenotype. CD8^+^HLA-DR^+^ Treg-induced suppression on CD8^+^ responder T cells was abrogated by an anti-PD1 neutralizing antibody. Anti-PD-1 did not abrogate the suppressor effect induced on responder CD4^+^ T cells. In addition, CD8^+^HLA-DR^+^ Treg induced a preferential death on responder CD8^+^ T cells. This effect was not reversed by PD-1 neutralization. After activation, most CD8^+^HLA-DR^+^ Treg acquire programmed death-ligand 1 (PD-L1) expression. Interestingly, PD-L1 may induce apoptosis through CD80 expressed on activated CD8^+^ responder T cells. After PBMCs stimulation, CD8^+^HLA-DR^+^ Treg cells showed an increased frequency of IFN-γ and TNFα positive cells and higher degranulation. These data strongly argue against CD8^+^HLA-DR^+^ Treg being exhausted cells. Overall, the data presented in this study indicate that CD8^+^HLA-DR^+^ Treg and CD4^+^FOXP3^+^ Treg share phenotypic and functional features, which may provide cues to similar involvements in the control of antitumor immune responses and autoimmunity.

## Introduction

Regulatory T cells (Treg) play a key role in maintaining immune homeostasis and in preventing the onset of autoimmune diseases (1). Although several subsets of Treg have been described, the best characterized is defined by expression of CD4, CD25, and the transcription factor forkhead box P3 (FOXP3) (2). Most circulating CD4^+^FOXP3^+^ Treg originate in the thymus (tTreg). However, naïve CD4^+^ T cells may also be induced to express FOXP3 in the periphery, representing a minority (pTreg) population (3) required for fetal tolerance (4).

We recently described a novel human regulatory CD8^+^HLA-DR^+^ T cell population present in adult and umbilical venous blood samples (5) representing a small subset in peripheral blood (PB) or cord blood mononuclear cells (CBMCs). The comparison between CD8^+^HLA-DR^−^ and CD8^+^HLA-DR^+^ T cells shows similar expression of the co-stimulatory molecule CD28, which differentiates them from the previously characterized CD8^+^CD28^−/low^ T suppressor cells originally generated *in vitro* by multiple rounds of T cell stimulation by allogenic APCs (6). Another natural CD8^+^ Treg population distinguished by expression of CD122 (7) was described in mice, but has not yet been identified in humans, and appear to exert their suppressor effect via IL-10.

The presence of CD8^+^HLA-DR^+^ Treg in cord blood strongly suggests that these Treg most likely originate from thymic emigrants and gradually increase over time. Their expansion is presumably induced through an encounter with environmental or self-antigens that generate the memory-like phenotype observed in adult CD8^+^HLA-DR^+^ Treg.

In the control of peripheral T-cell tolerance and autoimmunity, checkpoint pathways involving particularly cytotoxic T-lymphocyte–associated antigen 4 (CTLA-4) and programmed death 1 (PD-1) are thought to operate at different stages of an immune response (8), CTLA-4 acting at the initial stage of naïve T-cell activation, typically in lymph nodes (9). PD-1 pathway regulates previously activated T cells at later stages of immune response, primarily in peripheral tissues (8). Similarities and differences in these pathways have greatly contributed to cancer therapy involving immune checkpoint blockade (ICB).

In our previous study we identified features shared between CD8^+^HLA-DR^+^ Treg and classical CD4^+^FOXP3^+^ Treg cells; these included the requirement for cell-to-cell contact mainly involving CTLA-4, and complete abrogation of suppressor capacity by blocking this B7 ligand. In the present study we expanded phenotypic and functional characterization of CD8^+^HLA-DR^+^ Treg cells, including the complete phenotype of the CD8^+^HLA-DR^+^ Treg cells, their developmental stage, their exhaustion status, and similarities with canonical CD4^+^FOXP3^+^ Treg cells. In addition, we demonstrated that anti-PD-1 selectively abrogates the suppressor effect on CD8^+^ effector cells without affecting CD4^+^ effector cells.

## Materials and methods

### Ethics statement

This study was approved by the Investigation and Ethics Committee at the Hospital de Clínicas “Jose' de San Martin” and Hospital de Pediatría S.A.M.I.C. “Prof. Dr. Juan P. Garrahan” in accordance with the Declaration of Helsinki.

### Subjects

Peripheral blood (PB) mononuclear cells were obtained from healthy adult donors (HD), and cord blood (CB) samples from umbilical cord veins of full-term healthy neonates. None of the HD, neonates, or their mothers had any hereditary disorders, hematologic abnormalities, or infectious complications.

### Peripheral blood and cord blood mononuclear cell isolation

Freshly isolated PBMCs or CB mononuclear cells were isolated through Ficoll-Hypaque gradient centrifugation (GE Healthcare Life Sciences). After two washes with PBS, cells were suspended in RPMI medium.

### Antibodies, flow cytometry, and analysis of cytokine production

Isolated peripheral and cord blood mononuclear cells were incubated for 15 min at room temperature (RT) with fluorescence-conjugated mAbs purchased from the following sources: Biolegend: anti-CD3 (PerCP or Pacific Blue), anti-CD8 (APC-Cy7 or PerCP), anti-HLA-DR (FITC, PE or Brilliant Violet 421), anti-CD45RA (PE-Cy7), anti-CD27 (PE-Texas Red), anti-CD28 (PE or Brilliant Violet 711), anti-CCR7 (FITC or Brilliant Violet 785), anti-CCR5 (PE-Cy7), anti-CXCR3 (FITC), anti-CCR4 (Brilliant Violet 421), anti-PD-1 (PE or Brilliant Violet 711), anti-PD-L1 (APC), anti-CD155 (PE-Cy7), anti-Eomes (PE-Cy7), anti-CD127 (PE), anti-IFN-γ (PE-Cy7), anti-TNFα (Brilliant Violet 711), anti-CD107a (PE or FITC), anti-Ki-67 (PE or FITC). eBiosciences: anti-TIM-3 (APC), anti-CTLA-4 (PE), anti-TIGIT (PerCPeFluor710). Immunotools: anti-CD8 (APC), anti-HLA-DR (PE), anti-Granzyme B (FITC). For intranuclear staining, PBMCs were fixed and permeabilized with FOXP3 / Transcription Factor Fixation/Permeabilization Concentrate and Diluent solution (eBioscience) following the manufacturer's instructions. Anti-Ki-67 Ab was incubated after permeabilization. To detect intracellular cytokines, PBMCs were activated with PMA (50 ng/mL) and Ionomycin (1 μg/mL) for 4 h in the presence of monensin (Golgi stop-BD Biosciences). Alternatively, PBMCs were activated with plate-coated anti-CD3 (1 μg/mL) and soluble anti-CD28 (1 μg/mL). Anti-CD107a was added during *in vitro* stimulation to detect degranulation. After permeabilization using the BD Cytofix/Cytoperm Fixation/Permeabilization Kit (BD Biosciences), anti-IFN-γ, anti-TNFα, anti-CTLA-4, and anti-Granzyme B were added. LIVE/DEAD® Fixable Aqua (Thermofisher) or 7AAD (BD Biosciences) were used to exclude dead cells. The gating strategy is depicted in Figure S1. Flow cytometry was performed using FACS Aria II^u^ (BD Biosciences), and data analyzed using FlowJo software.

### Cell sorting

Fresh peripheral blood CD8^+^ T cells from healthy donors were enriched by negative selection using the RosetteSep Human CD8^+^ T Cells Enrichment Cocktail (StemCell Technologies) following manufacturer's protocol. Purified CD8^+^ T cells were stained with anti-CD8 APC and anti-HLA-DR PE antibodies (Immunotools) and sorted with a FACS Aria II^u^ flow cytometer (BD Biosciences), obtaining the following subsets: CD8^+^HLA-DR^+^ and CD8^+^HLA-DR^−^. Both subsets were collected into complete RPMI 1640 medium containing 50% FCS and washed before further experiments. The purity of each population, determined by flow cytometry, was always over 95%. For CD8^+^ cells depletion, peripheral blood mononuclear cells from healthy donors were isolated by density gradient, and CD8^+^ lymphocytes were depleted after staining with anti-CD8 APC (Immunotools). CD8^+^ depletion was ~99% effective.

### Suppression assay

Responder autologous PBMCs were labeled with 5 μM of CellTrace Violet (ThermoFisher) following manufacturer's protocol. 5 × 10^4^ responder cells were plated on 96 round-bottom wells and co-cultured in complete RPMI 1640 medium [Life Technologies] (supplemented with penicillin, streptomycin, L-glutamine [Sigma-Aldrich], and 10% FCS [Natocor]) with unlabeled highly purified CD8^+^HLA-DR^+^ or CD8^+^HLA-DR- T cells in a responder: suppressor ratio of 1:1. Cells were stimulated with 1 μg/mL of plate-coated CD3 (Biolegend) and 1 μg/mL of soluble CD28 (Biolegend). On day 4 post-activation, cells were harvested, and proliferation of CellTrace Violet-labeled cells was assessed by flow cytometry. At this time point, cells were stained with anti-human CD3 FITC (Immunotools), anti-human CD4 BV605 (Biolegend) and anti-human CD8 APC (Immunotools), and incubated with 7-AAD (BD Bioscience) to analyze cytotoxicity and exclude dead cells. Percentage of suppression was calculated by dividing the number of proliferating CellTrace Violet-diluting responder cells in the presence of suppressor cells by the number of proliferating responder cells when cultured alone, multiplied by 100. Unlabeled, stimulated, and unstimulated cells were used as controls.

### Neutralizing assay

To investigate neutralizing the suppressor effect, purified anti-PD-1 antibody (0.5 and 1 μg/mL; Biolegend) was added in the same culture conditions described for the suppression assay above, using the corresponding isotype-matched mAb as control. Purified PD-L1 neutralizing antibody (Biolegend) was used at 5 μg/mL. To test involvement of CTLA-4 added together with anti-PD-1 in the suppression mechanism, 5 μg/mL of anti-CTLA-4 (BD Biosciences) was used.

### Co-culture of CD8^+^-depleted PBMCs with highly purified CD8^+^HLA-DR^−^ or CD8^+^HLA-DR^+^ cells

Autologous 5 × 10^5^ CD8^+^-depleted PBMCs were plated on 48 multiwell plates in complete RPMI medium, and co-cultured with highly purified 0.9 × 10^5^ CD8^+^HLA-DR^−^ (5.5: 1), or 0.1 × 10^5^ CD8^+^HLA-DR^+^ (50: 1) T cells (the number of cells were defined according to their relative frequencies within PBMCs). Cells were activated with plate-coated aCD3 (1 μg/mL; Biolegend) plus soluble aCD28 (1 μg/mL; Biolegend). On day 4 post-activation, cells were harvested, and surface HLA-DR and intra-cellular Ki-67 were assessed by flow cytometry. Dead cells were excluded using LIVE/DEAD® Fixable Aqua (Thermofisher). Unstimulated cultures were used as negative controls.

### Activation of PBMCs and CBMC

Peripheral Blood (PB) or Cord Blood (CB) mononuclear cells were activated with plate-coated anti-CD3 (1 μg/mL) plus soluble anti-CD28 (1 μg/mL). In both cases, 5 × 10^5^ cells were plated on 48 multiwell plates in complete RPMI medium. For PBMCs kinetics experiments, cells were stained on day 4 after activation. For CB samples, cells were stained on day 2 after activation.

### Statistical analysis

The normality of variable distribution was assessed by the Kolmogorov- Smirnov goodness-of-fit test. Once the hypothesis of normality was accepted (*p* = 0.05), comparisons were performed using paired and unpaired Student *t*-tests, as appropriate. If the hypothesis of normality was rejected, analysis was performed using Wilcoxon's rank-sum test. A *p*-value of 0.05 was considered significant.

## Results

### CD8^+^ T cells expressing HLA-DR identified regulatory T cells expressing the checkpoint molecules CTLA-4 and PD-1

**Figure 1:**
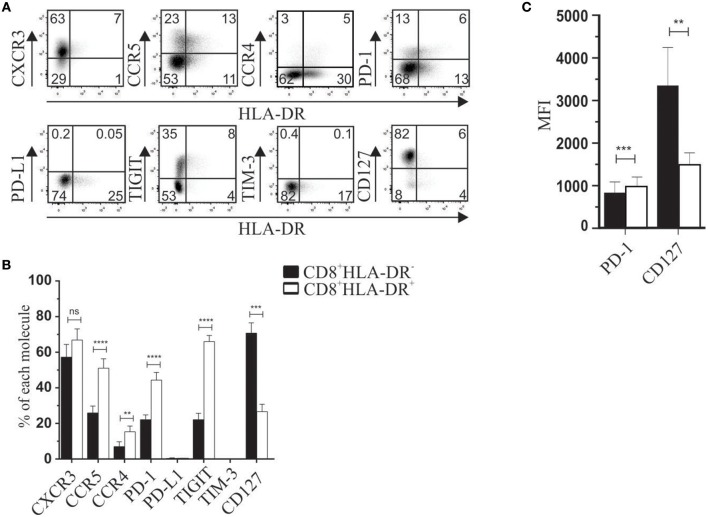
Phenotypic characterization of CD8^+^HLA-DR^+^ Treg and CD8^+^HLA-DR^−^ cells. **(A)** PBMC from healthy donors were isolated by density gradient, stained, and analyzed by flow cytometry. Dot plots are gated on total CD8^+^ T cells. **(B)** Graph shows comparative frequency of each molecule gated on CD8^+^HLA-DR^−^ and CD8^+^HLA-DR^+^ subsets. **(C)** Mean fluorescence intensity (MFI) of PD-1 and CD127 expression. Data is presented as mean ± SEM of PD-1 (*n* = 17), CXCR3 (*n* = 5), CCR5 (*n* = 15), CCR4 (*n* = 7), PD-L1 (*n* = 5), TIGIT (*n* = 13), TIM-3 (*n* = 5), CD127 (*n* = 6). Paired *t*-test. ^**^p < 0.01; ^***^p < 0.001; ^****^p < 0.0001.

We first analyzed phenotypic markers previously associated with regulatory subsets. For instance, PD-1 and TIGIT are known to be expressed in classical FOXP3^+^ Treg (10–12), the IL-7 receptor (CD127) is broadly used to isolate CD4^+^FOXP3^+^ Treg, and CXCR3, CCR5, and CCR4 are present in Treg cells in peripheral tissues: CD4^+^CD25^+^FOXP3^+^ (13, 14), and CD4^+^Tr1^+^ (15, 16). Considering that HLA-DR is used as an activation marker, we also analyzed TIM-3 and PD-L1 molecules. Compared to CD8^+^HLA-DR^−^ T cells, our results showed a higher frequency of PD-1 (*p* < 0.0001) and TIGIT (*p* < 0.0001), and down-modulation of CD127 frequency (*p* = 0.0009) (Figures 1A,B). In addition to increased frequency, PD-1 expression was also significantly higher (*p* = 0.0003) as reflected by increased mean fluorescence intensity (MFI). CD127 MFI was also lower in CD8^+^HLA-DR^+^ Treg than in CD8^+^HLA-DR^−^ cells (*p* = 0.0031) (Figure 1C). Interestingly, the frequencies of CCR5 (*p* < 0.0001) and CCR4 (*p* = 0.0038), were greatly increased in CD8^+^HLA-DR^+^ Treg cells (Figures 1A,B).

### Naïve cord blood CD8^+^HLA-DR^+^ T cells rapidly acquired the phenotype of mature peripheral blood CD8^+^HLA-DR^+^ treg cells after activation

**Figure 2:**
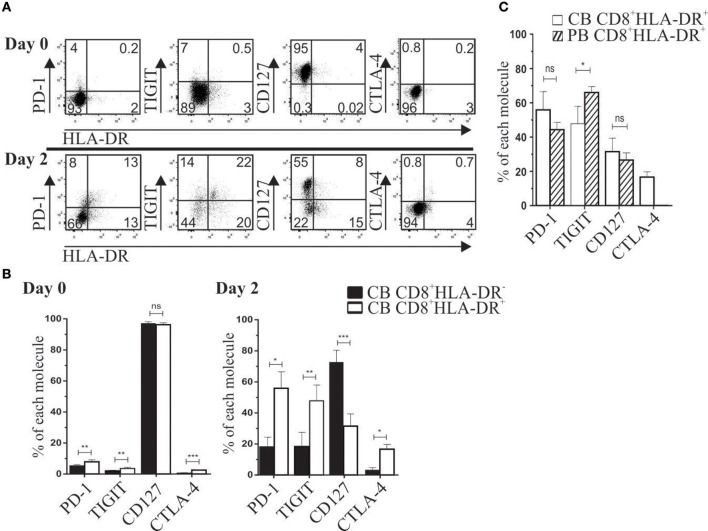
After TCR activation, cord blood CD8^+^HLA-DR^+^ Treg cells acquire a peripheral blood-like phenotype. (**A**) Representative dot plots show PD-1, TIGIT, CD127 and CTLA-4 expression after CB mononuclear cells activation for 2 days with plate-coated aCD3 (1 μg/mL) and soluble aCD28 (1 μg/mL). Analysis was performed on gated CD8^+^ cells at day 0 (Upper) and day 2 after activation (Lower). (**B**) Comparative frequency of PD-1, TIGIT, CD127 and CTLA-4 gated on CD8^+^HLA-DR^−^ and CD8^+^HLA-DR^+^ subsets, and presented as mean frequency ± SEM from six independent experiments. (**C**) Comparative frequency of CB and PB PD-1, TIGIT, CD127 and CTLA-4 on day 2 after activation (gated on each CD8 subset). Paired or unpaired *t*-test. ^*^p < 0.05; ^**^p < 0.01; ^***^p < 0.001.

In basal conditions, cord blood (CB) CD8^+^HLA-DR^−^ and CD8^+^HLA-DR^+^ subsets showed a naïve phenotype, with low frequency of PD-1, TIGIT, and CTLA-4 (< 10%), and more than 90% of cells from both subsets expressed the CD127 receptor (Figures 2A,B). Despite these similarities, compared to their CD8^+^HLA-DR^−^ counterpart, CD8^+^HLA-DR^+^ Treg showed a slightly but significantly increased number of cells expressing PD-1 (*p* = 0.0079), TIGIT (*p* = 0.0079), and CTLA-4 (*p* = 0.0003) (Figure 2B). Because we previously observed that cord blood CD8^+^HLA-DR^+^ Treg were able to suppress responder cell proliferation, we analyzed the expression kinetics of relevant molecules for suppression after activation by anti-CD3 plus anti-CD28 antibodies. As depicted in Figures 2B,C, as soon as 2 days after activation, CB CD8^+^HLA-DR^+^ acquired a phenotype similar in frequency to the one observed in mature peripheral blood CD8^+^HLA-DR^+^ Treg, including sharply decreased frequency of CD127 (*p* = 0.0002), increased frequency of PD-1 (*p* = 0.01), TIGIT (*p* = 0.0084), and CTLA-4 (*p* = 0.02), and preservation of the naïve phenotype. The ability of CB CD8^+^HLA-DR^+^ to rapidly acquire these regulatory molecules may explain the suppressor capacity of *ex vivo* CB CD8^+^HLA-DR^+^ we previously reported (5).

**Figure 3:**
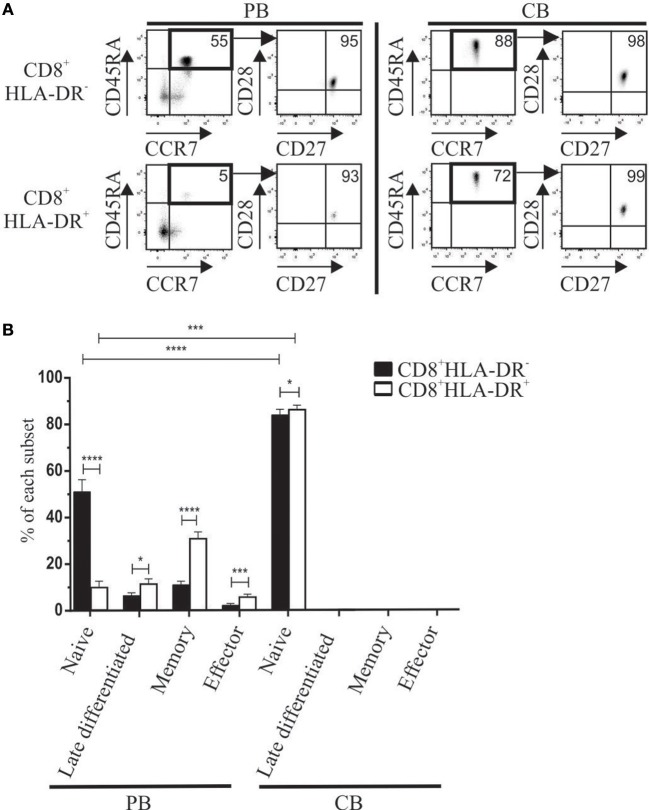
Differences in maturation status detected in CD8^+^HLA-DR^−^ and CD8^+^HLA-DR^+^ Treg cells from adult peripheral blood (PB) or cord blood (CB). **(A)** Representative dot plots show the expression of CD45RA vs. CCR7, and CD28 vs. CD27 used herein to define the maturation stage of peripheral blood (*n* = 13) (Left) and cord blood mononuclear cells (*n* = 6) (Right). Arrows indicate the sequence of the analysis. **(B)** Shows comparative frequency of each maturation stage between CD8^+^HLA-DR^−^ and CD8^+^HLA-DR^+^ in PB (*n* = 13) and CB (*n* = 6). Data is presented as mean frequency ± SEM. Paired and unpaired *t*-test were used when comparing results from the same donor or comparing PB and CB samples, respectively. ^*^p < 0.05; ^***^p < 0.001; ^****^p < 0.0001.

We also analyzed the developmental stage of CD8^+^HLA-DR^−^ and CD8^+^HLA-DR^+^ cells in peripheral blood (PB) and cord blood (CB) mononuclear cells. Meanwhile PB CD8^+^HLA-DR^−^ cells showed a higher frequency of naïve cells (CD45RA^+^CCR7^+^CD27^+^CD28^+^) (*p* < 0.0001), most CD8^+^HLA-DR^+^ Treg have an increased number of late differentiated cells (CD45RA^+^CCR7^−^CD27^−^CD28^−^) (*p* = 0.0317), memory (CD45RA^−^CCR7^+/−^CD27^+^CD28^+^) (*p* < 0.0001), and effector (CD45RA^−^CCR7^−^CD27^−^CD28^−^) (*p* = 0.0003) (Figures 3A,B). Conversely, as expected, the frequency of late differentiated, memory and effector T cells within cord blood were almost undetectable, being mostly CD8^+^HLA-DR^−^ and CD8^+^HLA-DR^+^ naïve cells (Figures 3A,B).

### Immunosuppressive properties of CD8^+^HLA-DR^+^ treg cells are associated with PD-1 molecule

**Figure 4:**
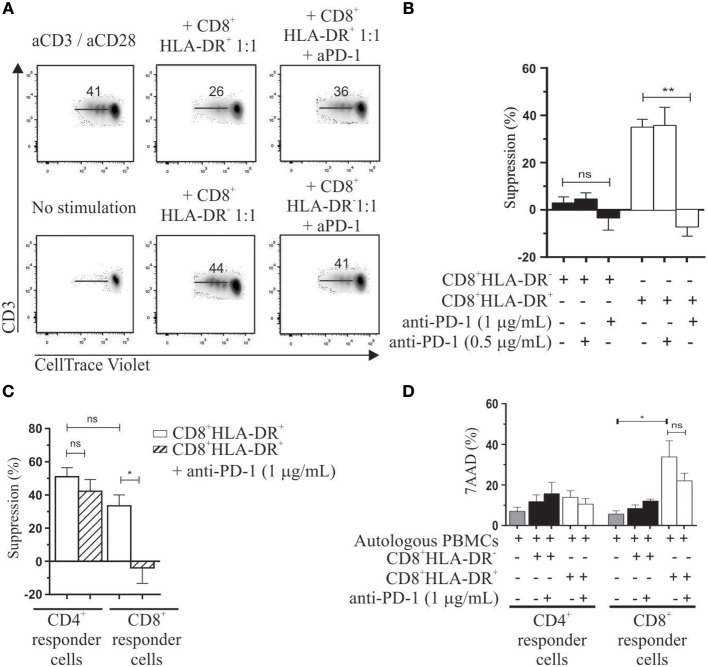
Immunosuppressive properties of CD8^+^HLA-DR^+^ Treg. Autologous PBMC labeled with CellTrace Violet were used as responder cells. CD8^+^HLA-DR^−^ and CD8^+^HLA-DR^+^ sorted cells were cultured with 5 × 10^4^ PBMC in a 1:1 ratio (suppressor: responder). After 4 days' activation with aCD3/aCD28 (1 μg/mL) proliferation was measured by CellTrace Violet dilution. **(A)** Representative dot plots from gated CD3^+^ cells showing the role of anti-PD-1 in reversing suppressor capacity of CD8^+^HLA-DR^+^ Treg cells. **(B)** Shows mean frequency of suppression induced by CD8^+^HLA-DR^+^ Treg and the reversion effect after addition of anti-PD-1 antibody (*n* = 5). **(C)** Similar to the analysis performed in **(B)** but analyzed on gated CD4^+^ or CD8^+^ T cells (*n* = 4). **(D)** Shows mean frequency of 7AAD positive cells at day 4 of the suppression assay (*n* = 5). Data is presented as mean ± SEM. Paired *t*-test. ^*^p < 0.05; ^**^p < 0.01.

Previously we reported the involvement of CTLA-4 in the suppressor mechanism of these cells (5). Given the high frequency and expression of PD-1 on CD8^+^HLA-DR^+^ Treg, we investigated the effect of an anti-PD-1 neutralizing antibody on the suppressor activity of this subset. We assessed the *in vitro* suppressive capacity of purified CD8^+^HLA-DR^−^ and CD8^+^HLA-DR^+^ T cells by diluting CellTrace Violet-labeled autologous PBMCs (hereafter called responder cells) co-cultured with each CD8^+^ T cell subset, and stimulated for 4 days with anti-CD3 plus anti-CD28 antibodies. Purified CD8^+^HLA-DR^+^ Treg were able to suppress the proliferative response of activated responder cells (Figure 4A). Anti-PD-1 antibody at 1 μg/mL fully abrogates the suppressor effect of CD8^+^HLA-DR^+^ Treg cells (*p* = 0.0015) (Figure 4B; Table 1). Similarly, an anti-PD-L1 neutralizing antibody induces the same effect (Figure S2). To further analyze the potential target population, we investigated the effect of adding CD8^+^HLA-DR^+^ to gated CD4^+^ or CD8^+^ CellTrace Violet-labeled responder cells. Addition of CD8^+^HLA-DR^+^ induced a decrease of over 50% in the proliferative capacity of CD4^+^ responder T cells and a relatively lower proliferative capacity of responder CD8^+^ cells. Strikingly, anti-PD-1 antibody only abrogates the suppressor effect induced on CD8^+^ responder cells (*p* = 0.0192) (Figure 4C; Table 2). To elucidate if the different suppressor behavior over CD8^+^ and CD4^+^ responder cells was associated to their PD-1 and PD-L1 expression, we analyzed the kinetics of these molecules on days 0 and 4 during the suppression culture. We observed that after activation PD-1 and PD-L1 increased in a similar way on both CD8^+^ and CD4^+^ responder cells (Figure S3). As also depicted in the same figure, CTLA-4 behaved similarly to PD-1 and PD-L1 on both responder subsets. These results suggest that the different effect observed on CD8^+^ and CD4^+^ responder cells is independent of their PD-1 or PD-L1 expression. However, when anti-CTLA-4 was added together with anti-PD-1, the suppressor effect on CD4^+^ responder cells was also abrogated (Figure S4). All together, these results suggest that CD8^+^HLA-DR^+^ Treg suppress both CD4^+^ and CD8^+^ responder populations, but only CD8^+^ proliferation is downregulated through PD-1 molecule expressed on CD8^+^HLA-DR^+^ Treg. However, the combined addition of both anti-PD-1 and anti-CTLA-4 were able to suppress also CD4^+^ responder cells. Of note, the addition of sorted CD8^+^HLA-DR^+^ (but not CD8^+^HLA-DR^−^) induced 37 ± 6.5% of 7AAD^+^ cells within responder CD8^+^ cells (*p* = 0.0327), with no significant effect on CD4^+^ cells. This effect was not reversed by the addition of the anti-PD-1 antibody (Figure 4D).

**Table 1:** CD8^+^HLA-DR^+^ Treg mechanism of action.

	**Suppression (Average ± SEM)**
CD8^+^HLA-DR^−^ (1:1)	2.9 ± 2.5
CD8^+^HLA-DR^−^ (1:1) + aPD-1 (1 μg/mL)	−3.4 ± 5.1
CD8^+^HLA-DR^+^ (1:1)	35 ± 3.4
CD8^+^HLA-DR^+^ (1:1) + aPD-1 (1 μg/mL)	−7 ± 3.8

**Table 2:** Suppression effect of CD8^+^HLA-DR^+^ Treg cells on CD4^+^ and CD8^+^ target cells.

	**Suppression (Average ± SEM) on target CD4^+^ T cells**	**Suppression (Average ± SEM) on target CD8^+^ T cells**
CD8^+^HLA-DR^+^ (1:1)	51 ± 5.3	33.4 ± 6.5
CD8^+^HLA-DR^+^ (1:1) + aPD-1 (1 μg/mL)	42 ± 7	−4 ± 9.2

**Figure 5:**
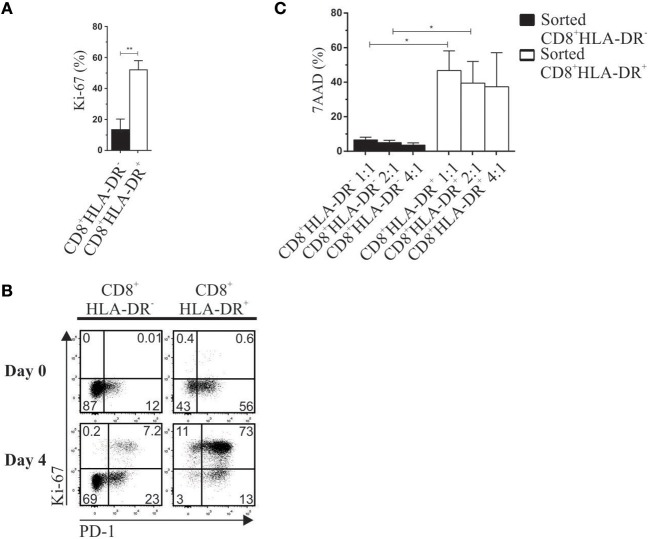
CD8^+^HLA-DR^+^ Treg have a high renewal rate. PBMC from healthy donors isolated by density gradient were activated with plate-coated aCD3 (1 μg/mL) and soluble aCD28 (1 μg/mL). Surface PD-1 and intracellular Ki-67 were stained on day 0 and 4 after activation. Ten minutes before reading the tubes in the flow cytometer, 7AAD death marker was added to the samples. **(A)** Mean frequency of Ki-67 on CD8^+^HLA-DR^−^ and CD8^+^HLA-DR^+^ cells after activation (*n* = 7). **(B)** Representative dot plots show the co-expression of PD-1 and Ki-67 gated on CD8^+^HLA-DR^−^ and CD8^+^HLA-DR^+^ subsets (*n* = 7). **(C)** Shows mean frequency of 7AAD 4 days after activation on sorted CD8^+^HLA-DR^−^ and CD8^+^HLA-DR^+^ cells, cultured with autologous PBMC (*n* = 5). Paired or unpaired *t*-test. ^*^p < 0.05; ^**^p < 0.01.

When PBMCs were stimulated for 4 days with anti-CD3 plus anti-CD28, and proliferation measured by Ki-67 expression, we observed that CD8^+^HLA-DR^+^ Treg cells showed a much stronger proliferative response (Figure 5A). Within these Ki-67^+^ proliferative cells, we detected the co-expression of PD-1 (Figure 5B). This co-expression was observed in 40 ± 15% of CD8^+^HLA-DR^+^ Treg. During the suppressor assay we also utilized 7-AAD to discriminate between dead and viable cells within the CellTrace Violet-negative sorted CD8^+^HLA-DR^−^ or CD8^+^HLA-DR^+^. Increased frequency of non-viable cells was detected in sorted CD8^+^HLA-DR^+^ cells: 40 vs. 5% in CD8^+^HLA-DR^−^ (irrespective of the ratio of responder and sorted CD8 cells; Figure 5C). These results suggest that CD8^+^HLA-DR^+^ Treg have an elevated renewal rate (high proliferation rate and high death rate).

### Expression of HLA-DR on highly purified CD8^+^HLA-DR^−^ and CD8^+^HLA-DR^+^ cells

**Figure 6:**
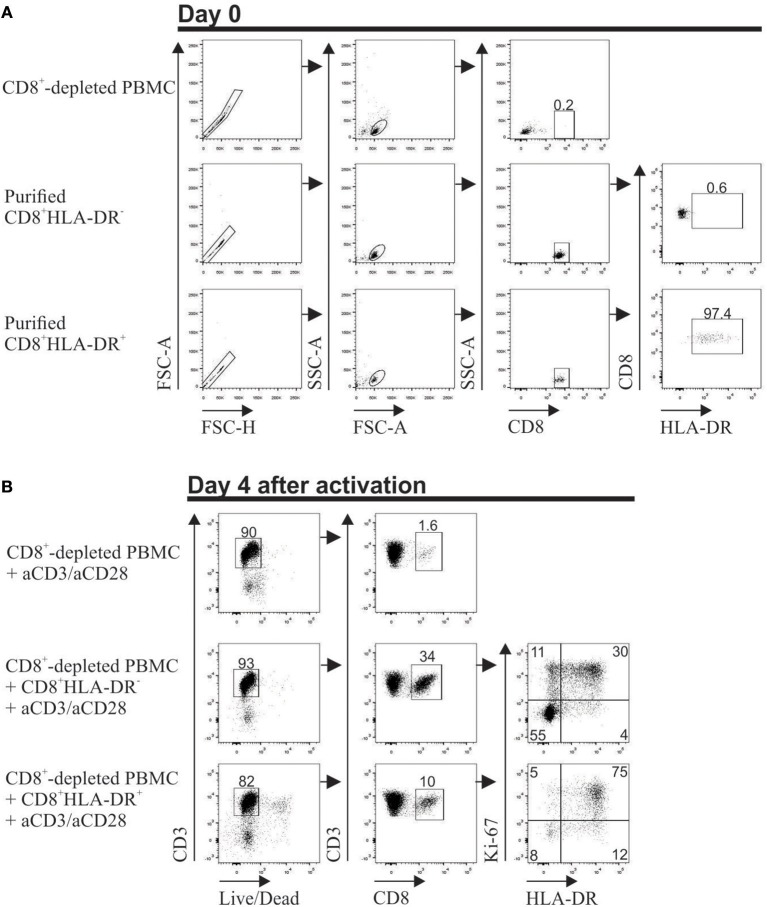
Stable expression of HLA-DR on *in vitro* activated CD8^+^HLA-DR^+^ Treg cells. PBMCs from healthy individuals were isolated, stained, and the following populations were purified in a FACS Aria II cell sorter: CD8^+^-depleted mononuclear cells, CD8^+^HLA-DR^−^ and CD8^+^HLA-DR^+^ subsets. **(A)** Dot plots show the purity of each population after sorting. **(B)** 5 × 10^5^ CD8^+^-depleted PBMCs were culture alone, or co-culture with purified 0.9 × 10^5^ CD8^+^HLA-DR^−^ or 0.1 × 10^5^ CD8^+^HLA-DR^+^ cells. On day 4 after aCD3 (1 μg/mL)/aCD28 (1 μg/mL) activation, surface HLA-DR and intra-cellular Ki-67 were stained. Singlets were excluded and living cells were selected by gating Live/Dead Aqua Fixable death marker-negative cells. HLA-DR and Ki-67 molecules were analyzed within gated CD8^+^ T cells.

To assess the stability of CD8^+^HLA-DR^+^ Treg cells, CD8^+^-depleted PBMCs were co-cultured with highly purified CD8^+^HLA-DR^−^ or CD8^+^HLA-DR^+^ cells. Additionally, HLA-DR and Ki-67 expression were evaluated after 4 days activation with anti-CD3 plus anti-CD28. Meanwhile 34% of CD8^+^HLA-DR^−^ cells acquire the HLA-DR molecule, almost all CD8^+^HLA-DR^+^ Treg cells remain HLA-DR^+^ (87%). Notably, when CD8^+^HLA-DR^−^ subset were co-cultured with the CD8^+^-depleted PBMCs 41% acquired Ki-67, that contrasted with the acquisition of Ki-67 by 80% of the CD8^+^HLA-DR^+^ cells (Figure 6). These results suggest that a subpopulation of CD8^+^HLA-DR^+^ Treg cells can be induced from CD8^+^HLA-DR^−^ after TCR activation.

### CD8^+^HLA-DR^+^ treg cells are not exhausted T cells

**Figure 7:**
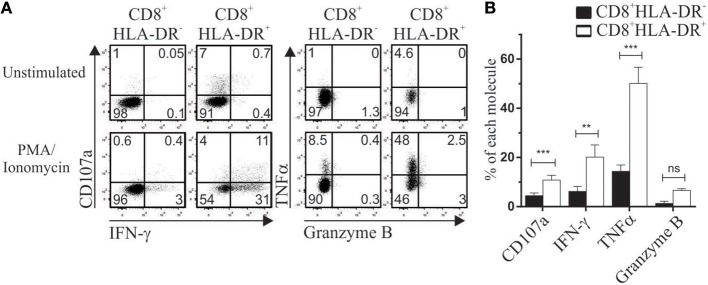
CD8^+^HLA-DR^+^ Treg are functionally competent cells. PBMC isolated by density gradient were stimulated for 4 h with PMA/Ionomycin in the presence of monensin. CD107a, IFN-γ, TNFα, and Granzyme B were stained and analyzed by flow cytometry. **(A)** Dot plots from one representative experiment showing expression of CD107a and IFN-γ (Left), TNFα and Granzyme B (Right) within CD8^+^HLA-DR^−^ and CD8^+^HLA-DR^+^ cells. **(B)** Graph shows the comparative frequency of each molecule gated on CD8^+^HLA-DR^−^ and CD8^+^HLA-DR^+^ subsets (*n* = 7). Data is presented as mean frequency ± SEM. Paired *t*-test. ^**^p < 0.01; ^***^p < 0.001.

The expression of PD-1, TIGIT and TIM-3 has been associated with exhausted cells (17, 18). As described in the previous section, CD8^+^HLA-DR^+^ Treg have an elevated renewal rate. Analysis of CD8^+^HLA-DR^+^ Treg within PMA/Ionomycin stimulated PBMCs showed that they had an increased number of cells expressing IFN-γ (*p* = 0.0098), TNFα (*p* = 0.0004), and CD107a (*p* = 0.0006). Moreover, no differences in secretion levels of granzyme B were detected when CD8^+^HLA-DR^−^ and CD8^+^HLA-DR^+^ subsets were compared (Figures 7A,B). Apart from their capacity to secrete cytokines and degranulate, these cells lack expression of the terminally exhausted marker TIM-3 (Figure 1A).

Given that PD-1 ligation can antagonize CD3 signaling, we evaluated the capacity of these Treg to secrete IFN-γ and degranulate in response to anti-CD3 plus anti-CD28 stimulation. Similarly to PMA/Ionomycin stimuli, a higher frequency of CD107a (*p* = 0.0178) and IFN-γ (*p* = 0.095) cells was observed in CD8^+^HLA-DR^+^ cells than in CD8^+^HLA-DR^−^ (Figure S5), suggesting that CD8^+^HLA-DR^+^ Treg are capable of responding to TCR ligation.

**Figure 8:**
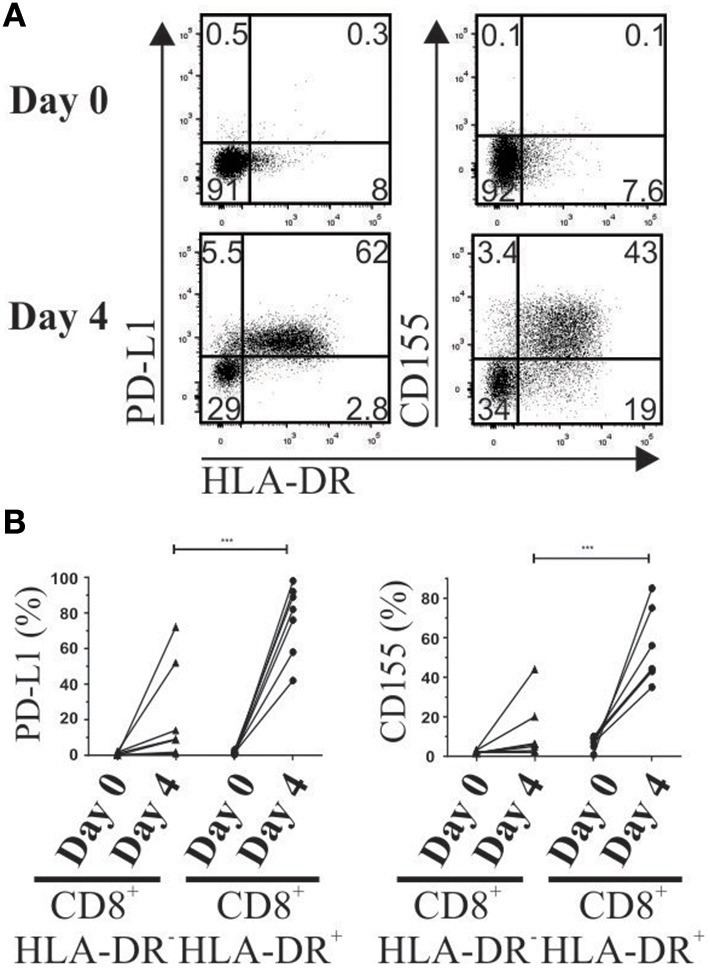
Increased expression of PD-1 and TIGIT ligands (PD-L1 and CD155) after T cell activation. Four days after PBMC activation, PD-L1 and CD155 were stained and analyzed by flow cytometry. (**A**) Representative dot plots show co-expression of HLA-DR, PD-L1 and CD155 gated on total CD8^+^ T cells on day 0 (Upper) and day 4 after activation (Lower). **(B)** Frequency of PD-L1 and CD155 gated on CD8^+^HLA-DR^−^ and CD8^+^HLA-DR^+^ subsets detected before and after 4 days' activation. Each point corresponds to an independent experiment (*n* = 7). Paired *t*-test. ^***^p < 0.001.

In basal conditions, total CD8^+^ T cells lack expression of PD-1 ligand 1 (PD-L1) and TIGIT ligand CD155. To mimic suppression assay conditions, PBMCs were activated with anti-CD3 plus anti-CD28, and PD-L1 and CD155 expression was evaluated on day 4 after activation. A higher frequency of both receptors (*p* = 0.0007 and *p* = 0.0008, respectively) was detected in activated CD8^+^HLA-DR^+^ Treg cells than in their CD8^+^HLA-DR^−^ counterpart (Figures 8A,B).

## Discussion

In our previous work (5) we showed that human CD8^+^HLA-DR^+^ but not CD8^+^HLA-DR^−^ cells suppress proliferation of autologous PBMCs responder cells through CTLA-4 co-inhibitory molecule. Our present data further characterize this regulatory subset by identifying a wide array of molecules showing preferential expression within CD8^+^HLA-DR^+^ Treg over CD8^+^HLA-DR^−^ cells. The complete phenotype includes, in addition to CTLA-4, expression of checkpoint receptors PD-1 and TIGIT and increased frequency of CCR4 and CCR5 chemokine receptors. Strikingly, this phenotype profile shared most of the molecules expressed by a subpopulation of CD4^+^FOXP3^+^ Treg cells with stronger regulatory capacity than conventional CD4^+^FOXP3^+^ Treg (11, 19). Low expression of CD127 and memory and effector-like phenotype are other features also shared by CD8^+^HLA-DR^+^ and CD4^+^FOXP3^+^ Treg cells (20).

Umbilical cord blood (CB) is a source of immature lymphocytes. CB CD8^+^ T cells are predominantly naïve cells defined by their CD45RA^+^CCR7^+^CD27^+^CD28^+^ co-expression. Concordantly with the similar naïve stage, both subsets showed low or absent frequency of CTLA-4, TIGIT and PD-1 molecules and high frequency of IL-7 receptor CD127. However, after only 2 days of TCR stimulation, cord blood CD8^+^HLA-DR^+^ cells rapidly acquired a phenotype quite similar to peripheral blood CD8^+^HLA-DR^+^ Treg cells.

The role of CTLA-4 as a mediator of suppressor function within classical CD4^+^FOXP3^+^ Treg cells is well known, as well as its capacity to negatively regulate immune responses (21). CTLA-4 is constitutively expressed in CD4^+^FOXP3^+^ Treg, probably because this molecule is a target of FOXP3 transcriptional factor (22). In contrast with the constitutive expression of PD-1 and TIGIT on CD8^+^HLA-DR^+^ Treg cells, CTLA-4 is induced after T cell activation, preferentially restricted to CD8^+^ cells expressing HLA-DR. Interestingly, and unlike PB CD8^+^HLA-DR^+^ Treg, umbilical CB CD8^+^HLA-DR^+^ showed augmented frequency of CTLA-4 in basal conditions, which may be explained by the slightly increased level of FOXP3 detected in CB HLA-DR^+^ Treg cells (5). Regarding PB CD8^+^HLA-DR^+^ Treg, our previous work showed that the regulatory effect is exerted by CTLA-4, and requires cell-cell contact. Therefore, blocking of CTLA-4 abrogated its suppressive effect in a dose-dependent manner (5).

PD-1 was first identified in 1992 (23). Initially it was believe that PD-1 triggered an inhibitory signal for CD3 signaling (24–26). Recent data support that CD28 costimulatory signaling is interrupted after PD-1 engagement rather than CD3 (27, 28), indicating that PD-1 suppresses T cell function primarily by inactivating CD28 signaling, playing a key role in regulating effector T cell function and responses to anti-PD-L1/PD-1 therapy. Thus, could be possible that the inactivation of CD28 costimulatory signaling may reproduce the suppressor effect triggered by PD-1. PD-1 expressed in CD4^+^FOXP3^+^ Treg cells plays a central role in regulating peripheral immune responses (10, 12). During chronic infection, CD4^+^FOXP3^+^ Treg that upregulate PD-1 showed a stronger suppressor function in mice infected with lymphocytic choriomeningitis virus (12). Within CD8^+^HLA-DR^+^ Treg cells, the high frequency of PD-1 strongly suggests that it may be associated with their regulatory capacity. In line with this, we found that an anti-PD-1 neutralizing antibody abrogated their suppressor activity. Multiple evidences seem to support that CTLA-4 and PD-1 receptors inhibit T cell activation by diverse mechanisms (26). Whereas, CTLA-4 increases the threshold needed for T cell activation by competing with CD28 co-receptor, PD-1 limits immune response at effector T cell activity level, preferentially in peripheral tissues where PD-L1 is expressed (8, 29, 30). Consequently, the immune checkpoint blockade by anti CTLA-4 and anti PD-1/PDL1 specific antibodies also differs in its mechanism of action (17).

An open question is whether manipulation of the PD-1/PD-L1 pathway is preventing priming or exhaustion, instead of reversing it. In the case of CD8^+^HLA-DR^+^ Treg cells, although similar suppressor activity on CD4^+^ or CD8^+^ responder cells was detected, we found that PD-1 neutralization specifically abrogates the suppression capacity on CD8^+^ responder cells with little effect on CD4^+^ responder cells, suggesting that PD-1 blockade may preferentially affect CD8^+^ T cells. Moreover, the similar behavior of PD-1, PD-L1, and CTLA-4 within CD4^+^ and CD8^+^ responder cells during the co-culture rise the possibility that a third population may be involved in their suppressor mechanisms. For instance, the interaction between CD8^+^HLA-DR^+^ Treg and APC during the suppression assay should be further addressed, because the lack of reversion of the suppressor effect on CD4^+^ responder cells could be related with the fact that APC activate naïve CD4^+^ T cells in the early phase of the immune response, typically in lymph nodes, while CD8^+^ T cells activation occurs later presumably improved by CD4^+^ T cells help. This explanation seems to be in line with a previous report of James Allison group (10) showing that anti- CTLA-4, but not anti-PD-1, acts at the initial stage of naïve T-cell activation, affecting CD4^+^ effector cells. Similarly, the same group reported that CD4^+^ T cells suffer a preferential expansion in CTLA-4(–/–) mice (31), and behave as key regulator in maintaining T cell homeostasis of CD4 vs. CD8 T cells (32). Therefore, it was expected that in the present study anti-CTLA-4 in contrast with anti-PD-1 abrogates the suppression on CD4^+^ effector cells. To our knowledge, this is the first time that PD-1 neutralization was shown to block the suppressor capacity of a CD8^+^ Treg population, acting specifically on CD8^+^ effector cells.

An additional feature of CD8^+^HLA-DR^+^ Treg cells is their high proliferative capacity associated with increased frequency of non-viable cells, meaning that they belong to a subset with a high renewal rate. In concordance with a previous study (33), we also found that highly proliferative CD8^+^HLA-DR^+^ showed a high frequency of PD-1^+^ cells. In addition, it was previously reported that CD4^+^CD45RO^+^FOXP3^+^CD25^hi^ T lymphocytes were highly proliferative, compared with memory CD4^+^CD45RO^+^FOXP3^−^CD25^−^ or naive CD4^+^CD45RA^+^FOXP3^−^CD25^−^ populations (34). Similarly, in inflamed skin CD4^+^ Treg were highly proliferative in comparison with conventional memory T helper cells (35). Of note, peripheral blood CD8^+^HLA-DR^+^ Treg with high proliferative rate also possess a memory-like phenotype. Regarding induced CD8^+^HLA-DR^+^ Treg cells, we also showed that a subset of highly purified CD8^+^HLA-DR^−^ T cells can acquire the expression of HLA-DR. This is in line with our previous report (5, 36), where we showed that induced CD8^+^HLA-DR^+^ Treg cells sorted after PBMCs activation behave as potent suppressor cells. The addition of highly purified CD8^+^HLA-DR^+^ cells to responder PBMCs not only induced suppression of proliferative CD3^+^ cells but also induced preferential death of responder CD8^+^ cells without any significant effect on CD4^+^ T cells. This effect was only slightly reversed after PD-1 neutralization, indicating that other mechanism/s may be involved in apoptosis induction. After activation, most CD8^+^HLA-DR^+^ Treg express PD-L1. In addition to PD-1, PD-L1 can bind to CD80 on activated CD8^+^ cells, triggering an apoptosis signal (37).

There is an emerging concept that not all FOXP3^+^ Treg are identical and may include distinct phenotypes specialized to selectively regulate specific effector T cell responses and control inflammation at defined anatomical tissue sites (38). For instance, TIGIT can inhibit T cell responses by binding the ligand CD155 on dendritic cells (DCs) thereby inhibiting IL-12 while inducing IL-10 production (39), or by directly inhibiting T cell activation and proliferation (40–42). Similarly to CD8^+^HLA-DR^+^ Treg cells, CD4^+^FOXP3^+^ cells with high suppressor function were also associated with TIGIT expression (43), CTLA-4, and PD-1 co-inhibitory molecules (39, 40), and within CD4^+^ TILs almost exclusively detected on FOXP3^+^ Treg (11). CD8^+^HLA-DR^+^ Treg also showed expression of CD155 TIGIT ligand. The agonistic effect of the anti-TIGIT antibody we used prevented us from observing abrogation of suppression by this antibody.

The expression of chemokine receptors may identify different human Treg cell subsets (44, 45). Another important similarity between CD4^+^FOXP3^+^ and CD8^+^HLA-DR^+^ Treg is the expression of CCR4, previously associated with stronger suppressor activity (43). In our case, CD8^+^HLA-DR^+^CCR4^+^ Treg cells showed augmented frequency and expression of PD-1 (data not shown). In line with the elevated expression of CCR4 and PD-1 on Treg cells, it was reported that treatment of T cells with anti-CCR4 immunotherapy enhance not only CD4 but also CD8 immune responses (46).

Exhausted T cells can be distinguished from T cell anergy or senescence (47, 48). Anergy occurs during T cell priming by inadequate costimulatory signals whereas senescence is growth arrest after extensive proliferation. In contrast, exhausted T cells arise from cells which initially gained effector function but were gradually silenced due to continuous TCR stimulation by a persistent antigen (49). High PD-1 expression is a hallmark of “exhausted” T cells that have experienced high levels of stimulation or reduced CD4^+^ T-cell help (18) as seen during chronic infections and cancer. The principal characteristic of exhausted T cells is loss of function in a hierarchical manner. IL-2 production and *ex vivo* killing capacity are lost at the early stage of exhaustion (18). Whereas, TNFα production is lost at an intermediate stage, IFN-γ is lost at the advanced stage of exhaustion (50). After a single round of anti-CD3 plus anti-CD28 activation, cord blood CD8^+^HLA-DR^+^ cells acquire most of the inhibitory markers associated with exhausted T cells while retaining their naïve phenotype. Therefore, it was inappropriate to associate this Treg subset with exhausted cells. Also, CD8^+^HLA-DR^+^ lack TIM-3 expression, and after PBMCs stimulation showed increased frequency of IFN-γ and TNFα positive cells and greater degranulation as revealed by CD107a expression. In addition, granzyme B (GzmB), increased in exhausted T cells (51), showed no differences between CD8^+^HLA-DR^−^ and CD8^+^HLA-DR^+^ T cells. All together, these data strongly argue against CD8^+^HLA-DR^+^ Treg being exhausted T cells.

Regarding biological implications of CD8^+^HLA-DR^+^ cells, we observed that these Treg cells were present in TILs of patients with NSCLC at even higher frequencies than CD4^+^FOXP3^+^ Treg cells (5). These results are supported by a report that TIGIT and PD-1 were expressed by a large percentage of NSCLC-infiltrating CD8^+^ T cells (52).

Overall, data presented in this study indicates that CD8^+^HLA-DR^+^ Treg and CD4^+^FOXP3^+^ Treg share phenotypic and functional features, and that they may be similarly involved in the control of antitumor immune responses and autoimmunity. Therefore, therapeutic strategies that aim to overcome Treg activity as a means of enhancing antitumor immune responses should integrate this novel intratumoral subset of highly suppressive CD8^+^HLA-DR^+^ Treg cells.

## Author contributions

AM and LF have made substantial contributions to the conception and design, the acquisition, analysis and interpretation of the data, and wrote the paper. LF also obtained funding. SB, LB, and PB performed experiments, and help with the analysis and interpretation of data.

### Conflict of interest statement

The authors declare that the research was conducted in the absence of any commercial or financial relationships that could be construed as a potential conflict of interest.
