# Supplementary figures and images for: Human CD8+HLA-DR+ Regulatory T Cells, Similarly to Classical CD4+Foxp3+ Cells, Suppress Immune Responses via PD-1/PD-L1 Axis
Source: Front Immunol. 2018 Nov 29;9:2788. doi: 10.3389/fimmu.2018.02788 (PMC6281883; doi:10.3389/fimmu.2018.02788)

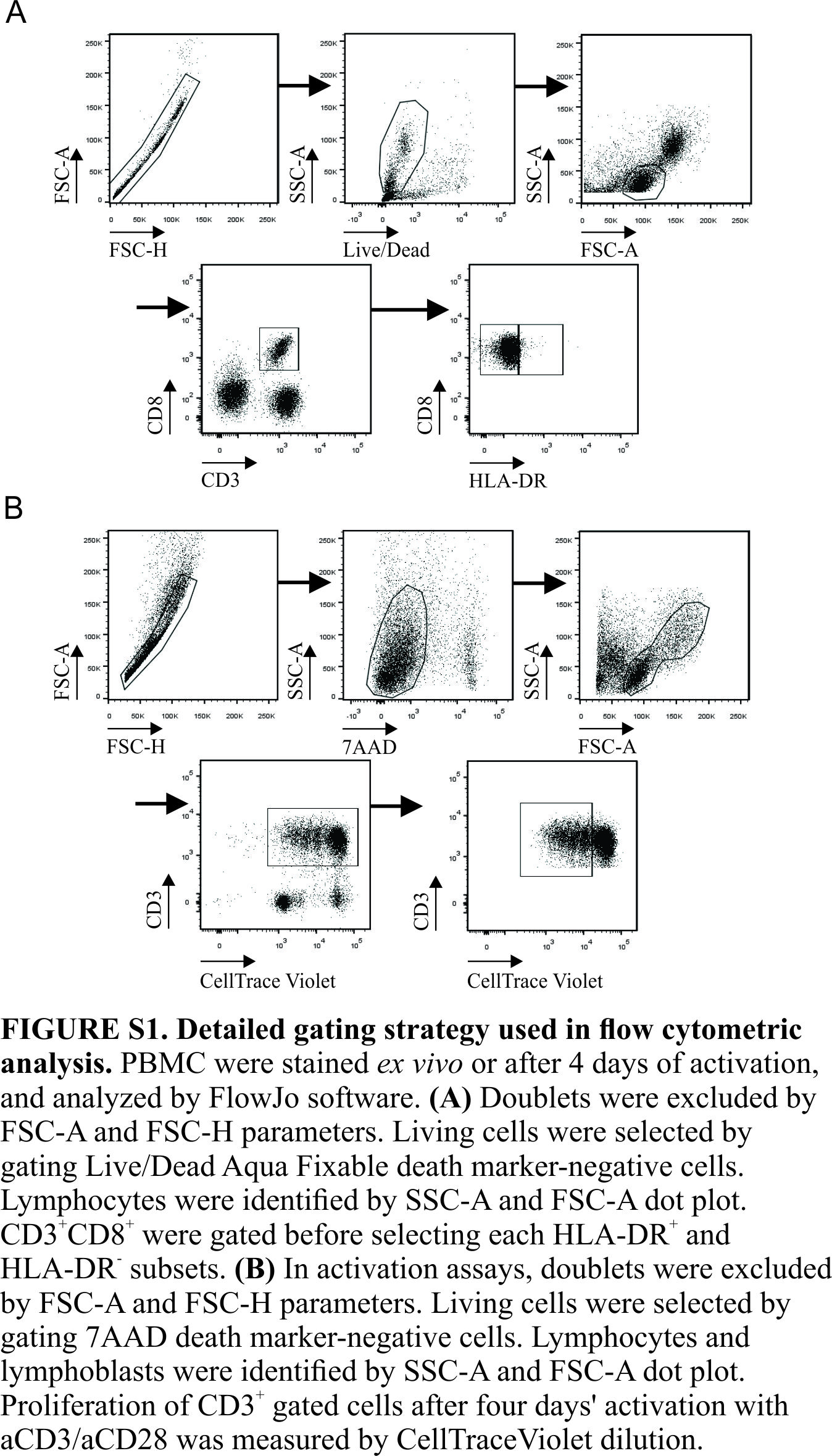

Supplement: Supplementary file 1 [file Image_1.jpeg]

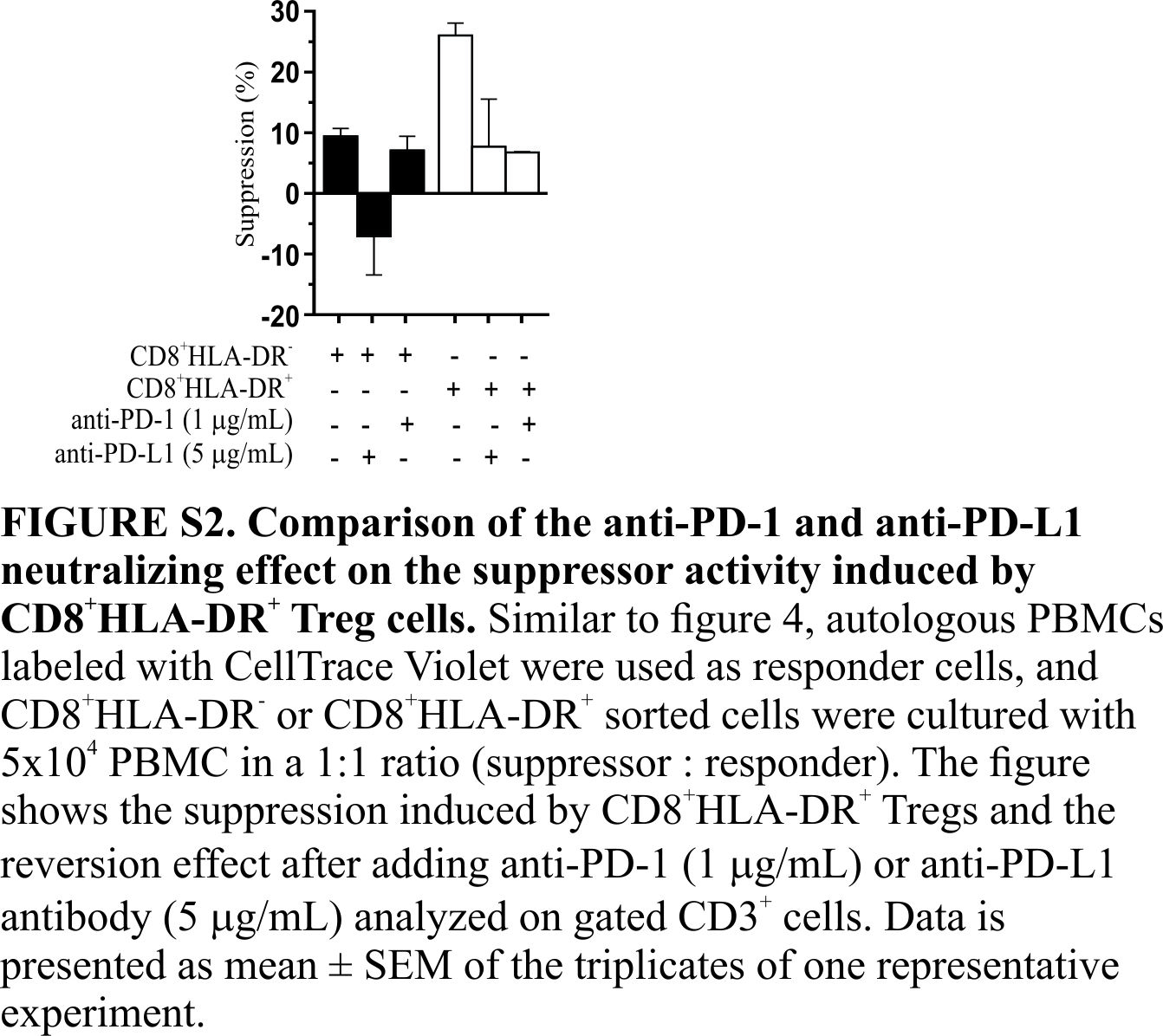

Supplement: Supplementary file 2 [file Image_2.JPEG]

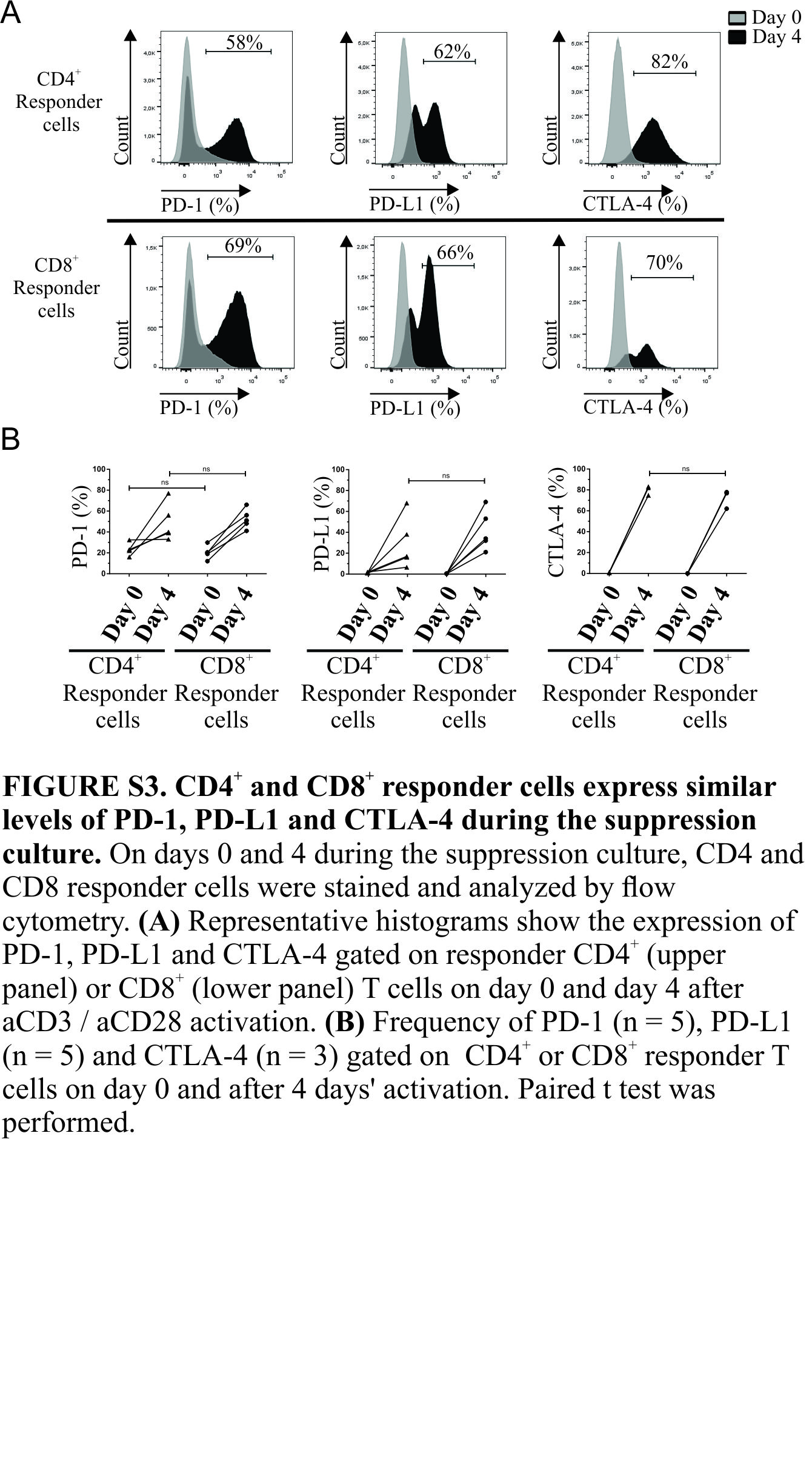

Supplement: Supplementary file 3 [file Image_3.JPEG]

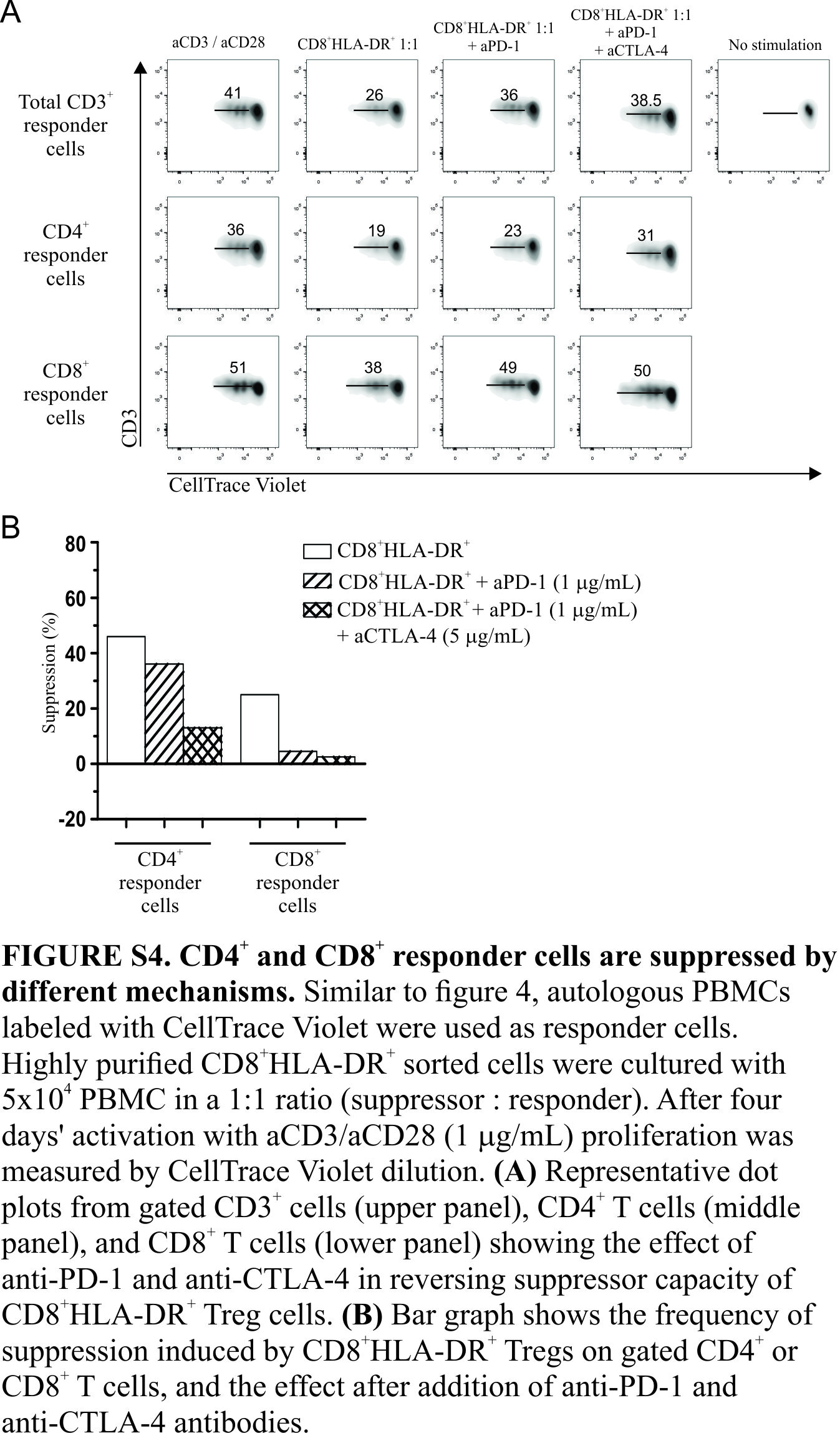

Supplement: Supplementary file 4 [file Image_4.JPEG]

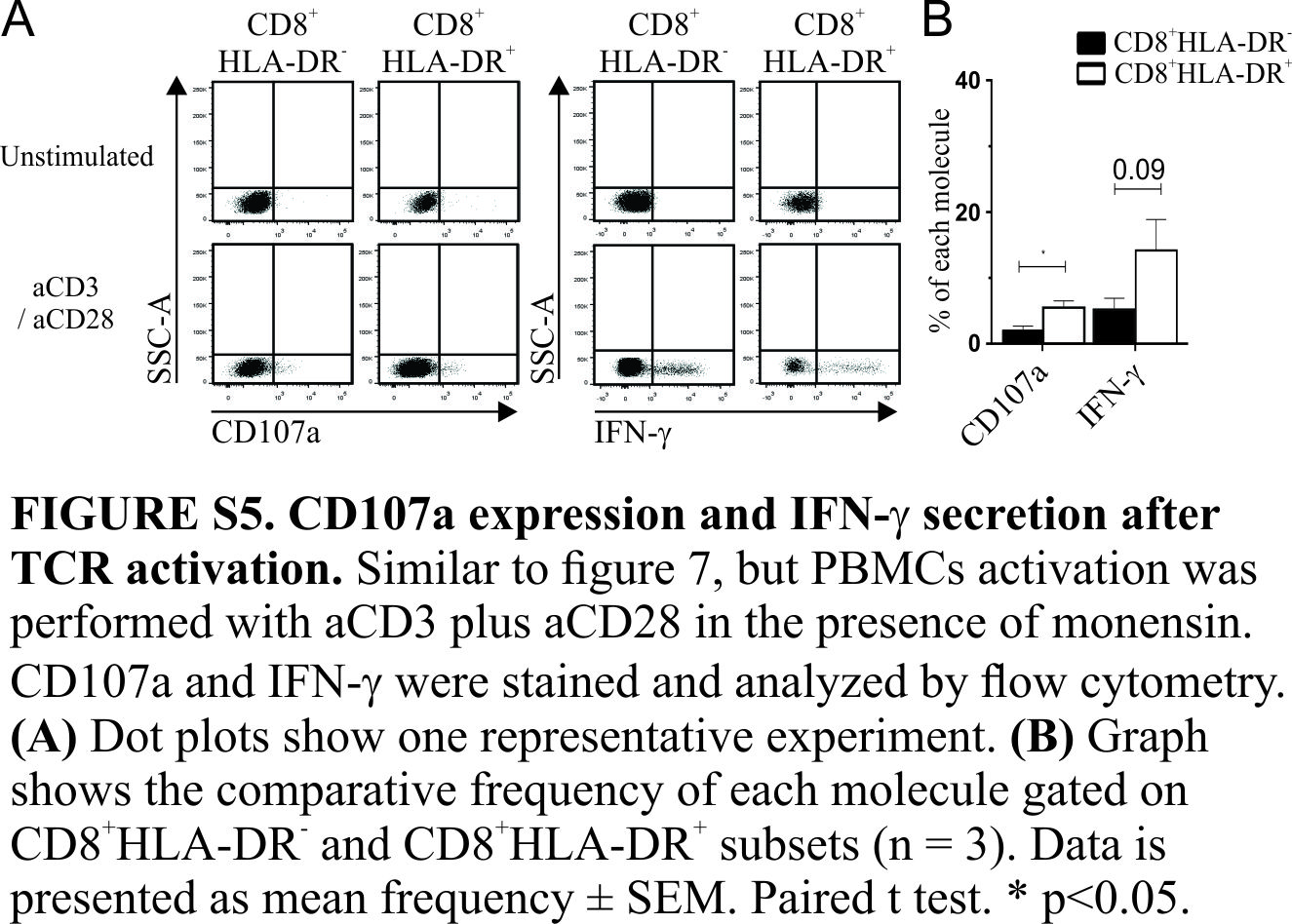

Supplement: Supplementary file 5 [file Image_5.JPEG]
